# Modification of Polyethylene Glycol-Hydroxypropyl Methacrylate Polymeric Micelles Loaded with Curcumin for Cellular Internalization and Cytotoxicity to Wilms Tumor 1-Expressing Myeloblastic Leukemia K562 Cells

**DOI:** 10.3390/polym16070917

**Published:** 2024-03-27

**Authors:** Siriporn Okonogi, Chuda Chittasupho, Tanongsak Sassa-deepaeng, Nattakanwadee Khumpirapang, Songyot Anuchpreeda

**Affiliations:** 1 Department of Pharmaceutical Sciences, Faculty of Pharmacy, Chiang Mai University, Chiang Mai 50200, Thailand; chuda.c@cmu.ac.th; 2 Center of Excellent in Pharmaceutical Nanotechnology, Faculty of Pharmacy, Chiang Mai University, Chiang Mai 50200, Thailand; 3 Agricultural Biochemistry Research Unit, Faculty of Sciences and Agricultural Technology, Rajamangala University of Technology Lanna Lampang, Lampang 52000, Thailand; tanongsaks@rmutl.ac.th; 4 Department of Pharmaceutical Chemistry and Pharmacognosy, Faculty of Pharmaceutical Sciences, Naresuan University, Phitsanulok 65000, Thailand; nattakanwadeek@nu.ac.th; 5 Department of Medical Technology, Faculty of Associated Medical Sciences, Chiang Mai University, Chiang Mai 50200, Thailand

**Keywords:** nanocarrier, cellular internalization, leukemic K562, antiproliferation, normal red blood cells, normal peripheral mononuclear cells

## Abstract

Curcumin loaded in micelles of block copolymers of ω-methoxypoly(ethylene glycol) and N-(2-hydroxypropyl) methacrylamide modified with aliphatic dilactate (CD) or aromatic benzoyl group (CN) were previously reported to inhibit human ovarian carcinoma (OVCAR-3), human colorectal adenocarcinoma (Caco-2), and human lymphoblastic leukemia (Molt-4) cells. Myeloblastic leukemia cells (K562) are prone to drug resistance and differ in both cancer genotype and phenotype from the three mentioned cancer cells. In the present study, CD and CN micelles were prepared and their effects on K562 and normal cells were explored. The obtained CD and CN showed a narrow size distribution with diameters of 63 ± 3 and 50 ± 1 nm, respectively. The curcumin entrapment efficiency of CD and CN was similarly high, above 80% (84 ± 8% and 91 ± 3%). Both CD and CN showed suppression on WT1-expressing K562 and high cell-cycle arrest at the G2/M phase. However, CD showed significantly higher cytotoxicity to K562, with faster cellular uptake and internalization than CN. In addition, CD showed better compatibility with normal red blood cells and peripheral blood mononuclear cells than CN. The promising CD will be further investigated in rodents and possibly in clinical studies for leukemia treatment.

## 1. Introduction

Leukemia is a cancer of the blood characterized by the accumulation of uncontrolled proliferation of abnormal blood cells. Recent statistics reported that leukemia is one of the five leading types of cancer death in patients younger than 40 years [1]. Treatment of leukemia is generally performed by chemotherapy, radiation therapy, and stem cell transplantation surgery. However, current chemotherapy is often restricted by dose-limiting toxicity and severe side effects [2]. Moreover, many patients fail to respond to the current chemotherapy. Some of them show incomplete responses or relapses that are caused by drug resistance after the treatment. Therefore, there is an urgent need to find alternative therapies. The investigation of new substances, especially those from natural resources, and particularly edible plants, becomes much more attractive due to their potential for anticancer activity, lower possibility of drug resistance, and lack of serious side effects on normal cells [3]. Curcumin is a natural phenolic compound extracted from the rhizomes of an edible plant, turmeric (*Curcuma longa*). This compound has been reported to have strong anti-oxidative, anti-inflammatory, and anti-septic properties [4]. In addition, curcumin can interfere with blood sugar. Many reports have shown that curcumin possesses anti-diabetic activity [5]. Curcumin supplementation can significantly reduce fasting blood glucose and hemoglobin A1C levels and other metabolic parameters in type 2 diabetes [6,7]. An in vitro study on cancer cell lines demonstrated that curcumin can inhibit cell-cycle progression and metastasis through many signaling pathways [8]. The previous in vitro study has shown that curcumin possessed strong inhibition on EoL-1 leukemic cells by induction of cell-cycle arrest [9]. However, curcumin has a problem of low aqueous solubility [10]. This poor property leads curcumin to have a limitation in clinical applications. A harmful organic solvent such as dimethyl sulfoxide (DMSO) is mostly used as a solvent to prepare curcumin solution for in vitro tests, whereas curcumin in a solid powder form is often used in in vivo studies. This powder form, in addition to the poor solubility of curcumin, is the cause of its very low bioavailability, resulting in suboptimal blood concentrations to achieve therapeutic effects. It was reported that 500 mg/kg orally administered curcumin gave a maximum plasma concentration of 0.06 ± 0.01 μg/mL, indicating that oral bioavailability was only 1% [11]. Previous studies in cancer patients demonstrated that curcumin did not present cytotoxicity at doses of up to 8 g/day; however, beyond 8 g/day, the bulky volume of the drug became unacceptable to patients [12]. Therefore, it is essential to improve the poor biopharmaceutical properties of curcumin. Nanocarriers have been used to increase efficiency and maintain sufficient drug concentration for a desirable period. During the last decades, various types of nanocarriers have been investigated. Among them, polymeric micelle is of the most interest because of its high efficiency in increasing the aqueous solubility of hydrophobic active compounds [13].

Polymeric micelles can be easily prepared from polymers having structures composed of both hydrophilic and hydrophobic chains. Various biodegradable polymers including amphiphilic peptides can serve as excellent polymers to form polymeric micelles for a single drug delivery, or in more advanced usages, as a multifunctional intelligent system [14,15]. Polymeric micelles can be designed in various strategies to improve efficacy and specificity for achieving targeted drug delivery and enhancing the therapeutic outcomes for patients [16], and have shown great promise in the targeted delivery of many anticancer agents [17,18,19]. Importantly, some pharmaceutical polymeric micelles have been proven and entered clinical evaluations [20,21,22]. Concerning curcumin, several kinds of polymeric micelles have been used to deliver this compound [23,24,25]. Loading of curcumin into polymeric micelles of poloxamer 407 was previously reported and the obtained micelle size was too big (182–415 nm) with a wide size distribution, leading to an unsuccessful delivery process [26]. Block copolymers composed of N-(2-hydroxypropyl) methacrylamide (HPMA) have been reported to possess good biocompatibility, non-immunogenicity, and possibilities for chemical functionalization [27]. Recently, HPMA with stimuli-responsive properties, such as pH and thermal sensitivity, has been reported [28,29]. HPMA dilactate and ω-methoxypoly(ethylene glycol) (PEG) undergo a simple self-association after heating above the critical micellization temperature (50 °C) to yield polymeric micelles, which were used to solubilize various hydrophobic compounds like paclitaxel [30], Si(sol)_2_Pc [31], and magnetic resonance imaging contrast agents [32]. In addition, it has been reported that the introduction of aromatic groups in the HPMA block copolymer can improve the loading capacity and drug retention for paclitaxel and docetaxel [33]. To extend the application of this block copolymer, in 2015, our group prepared curcumin-loaded HPMA-based polymeric micelles and reported that the obtained micelles had cytotoxicity against human ovarian carcinoma cells (OVCAR-3), human colorectal adenocarcinoma cells (Caco-2), and human acute lymphoblastic leukemia cells (Molt-4) [34], but we did not examine in K562 cell line. The three cell lines (OVCAR-3, Caco-2, and Molt-4) are completely different from K562 in both cancer genotype and phenotype. Thus, their cytotoxic effects may be different when compared to the K562 cell line. Although Molt-4 cells are leukemia cells, they are different from K562 cells because Molt-4 cells are lymphoblastic cell type and obtained from acute leukemia patients, whereas K562 cells are myeloblastic cell type and found in chronic myelocytic leukemia patients. In addition, the K562 cell line is a cell suspension type that differs from those of two adherent cell lines (OVCAR-3 and Caco-2). Taken together, the K562 cell line is different from the OVCAR-3, Caco-2, and Molt-4 cell lines. It is possible to show the different cytotoxicity. In addition, the compatibility of innovative nanomedicines with normal cells is one of the most important issues to be addressed. To the best of our knowledge, the effects of these polymeric micelles on human normal cells have not yet been reported. In addition, cytotoxicity with deep detail on the mechanism of action in cellular levels of curcumin-loaded micelles against K562 myeloblastic leukemia cells has not been well studied or reported elsewhere.

In the present study, we focused on the cytotoxicity of modifications of PEG-HPMA polymeric micelles loaded with curcumin against myeloblastic leukemia K562 cells and human normal cells. The polymeric micelles of HPMA-based block copolymers composed of ω-methoxypoly(ethylene glycol) and N-(2-hydroxypropyl) methacrylamide (PEG-HPMA) were formulated. The obtained micelles were modified to yield two types of micelles. One was modified by aliphatic dilactate (DL) substitution, leading to the acquisition of a thermosensitive copolymer of ω-methoxy poly(ethylene glycol)-b-(N-(2-hydroxypropyl) methacrylamide dilactate (PEG-HPMA-DL), and the other was modified by aromatic benzoyl (Bz) substitution, leading to the yield of a thermostable copolymer of ω-methoxy poly(ethylene glycol)-b-(N-(2-benzoyloxypropyl) methacrylamide (PEG-HPMA-Bz). Curcumin was loaded into the micelles of both polymers to obtain curcumin-loaded PEG-HPMA-DL (CD) and curcumin-loaded PEG-HPMA-Bz (CN). The cytotoxic effects of CD and CN on leukemia K562 cells and on human normal red blood cells (RBCs) as well as normal peripheral blood mononuclear cells (PBMCs) were compared. The mechanism of the cytotoxic action of the micelles on cellular responses in K562 proliferation, including cellular internalization and the induction of cell-cycle arrest, was deeply investigated. Furthermore, it is known that Wilms’ tumor 1 protein (WT1), a 48–52 kDa nuclear transcriptional activator or tumor repressor protein, is necessary for the induction of cell proliferation and differentiation [35]. The overexpression of WT1 can be used as a golden standard marker for the monitoring of acute and chronic myeloid leukemia or myeloproliferative syndrome [36]. In the current study, the WT1 protein was used as a target protein model to confirm the antileukemic activity of curcumin after loading into both modified polymeric micelles.

## 2. Materials and Methods

### 2.1. Materials

PEG, HPMA, curcumin, acetone, dimethyl formamide (DMF), dimethyl sulfoxide (DMSO), Ficoll-Paque solution, Tris (hydroxymethyl) aminomethane hydrochloride (Tris-HCl), potassium chloride (KCl), 3-(4,5-dimethylthiazolyl-2)-2,5-diphenyl tetrazolium bromide (MTT), RIPA buffer (containing 50 mM Tris-HCl, 150 mM sodium chloride (NaCl), 1% Triton X-100, 0.5 mM ethylenediaminetetraacetic acid, 0.1% sodium dodecyl sulfate, and protease inhibitor cocktail), SDS-polyacrylamide gel electrophoresis (SD-SPAGE), and glyceraldehyde-3-phosphate dehydrogenase (GAPDH) were from Sigma-Aldrich (St. Louis, MO, USA). K562, a potent leukemia cell line derived from chronic myelogenous leukemia patients in blast crisis, was purchased from the RIKEN BRC Cell Bank (Ibaraki, Japan). RPMI 1640 medium, fetal bovine serum, penicillin, and streptomycin were from Invitrogen™ Life (Carlsbad, CA, USA). Propidium iodide (PI) was from US Biological (Swampscott, MA, USA). Goat anti-rabbit IgG conjugated with HRP was from the Promega Corporation, (Madison, WI, USA). Luminata^TM^ Forte Western HRP Substrate was from the Millipore Corporation, (Billerica, MA, USA). Primary rabbit polyclonal anti-WT1 and rabbit polyclonal anti-GAPDH were from Santa Cruz Biotechnology (Santa Cruz, CA, USA). Other chemicals and solvents were of the highest grade available.

### 2.2. Preparation of Polymeric Micelles

PEG-HPMA-DL and PEG-HPMA-Bz were synthesized and characterized using ^1^H NMR spectroscopy and gel permeation chromatography as described previously [33]. The ^1^H NMR spectra were recorded using a Gemini 300 MHz spectrometer (Varian Associates Inc. NMR Instruments, Palo Alto, CA, USA), using d^6^-DMSO as a solvent and the DMSO peak at 2.50 ppm was used as the reference line. Gel permeation chromatography was performed using two serial PLgel 5 µm MIXED-D columns (Polymer Laboratories). Calibration was conducted by PEGs of different molecular weights and with narrow molecular weight distribution. The molecular weight of the PEG used was 5000 g/mol. The average molecular weights of PEG-HPMA-DL and PEG-HPMA-Bz, determined by gel permeation chromatography, were 27 and 28 kDa, with polydispersity indices of 1.7 and 1.3, respectively. The CD was prepared by the fast heating method as previously described with minor modifications [30]. Briefly, 10 mg/mL PEG-HPMA-DL solution in 100 mM phosphate buffer solution (PBS) was prepared and incubated overnight in an ice bath. Next, 100 μL of 10 mg/mL curcumin in acetone was added to 900 μL of the polymer solution at 0–4 °C. The micelles were formed by heating the mixture to 50 °C in a water bath for 1 min. The mixture was slowly cooled to room temperature. Blank PEG-HPMA-DL micelles (BD) were prepared in the same manner as CD but without adding curcumin. CN was prepared by the nanoprecipitation method. Firstly, 20 mg PEG-HPMA-Bz and 2 mg curcumin were dissolved in 500 μL of acetone. The mixture was slowly dropped into 2 mL of 100 mM PBS under stirring for 2 h until acetone was completely evaporated. Blank PEG-HPMA-Bz micelles (BNs) were prepared in the same manner as CN but without adding curcumin. The removal of free or non-entrapped curcumin from each CD and CN was performed by centrifugation. Briefly, the mixture of the freshly prepared curcumin-loaded polymeric micelles was added to a 1-mL microtube and centrifuged at 5000 rpm for 20 min. The non-entrapped and precipitated curcumin was spun down to the bottom of the microtube. The supernatant (micellar dispersion) was separated from the precipitates and filtered through a 0.20 μm membrane (Phenex™, Phenomenex Inc., Torrance, CA, USA) to remove some remaining free curcumin as tiny insoluble particles. The filtrate was used for further investigation. The curcumin content of the CD and CN was determined using a UV-Vis spectrophotometer (UV-2450, Shimadzu, Japan) at 425 nm by diluting 10 μL of CD or CN with 1990 μL of DMF. The reference solution in the reference cell was prepared by diluting 10 μL of blank micelles in 1990 μL of DMF to avoid the interference of the polymer. Curcumin solution in DMF (0.3–10.0 μg/mL) was used for calibration. Drug entrapment efficiency (EE) and drug loading capacity (LC) were calculated using Equations (1) and (2), respectively:

(1) $$EE(\%)=\frac{A_{E}}{A_{C}}\times 100$$

(2) $$LC(\%)=\frac{A_{E}}{A_{P}}\times 100$$

where *A_E_* is the amount of the entrapped curcumin (mg/mL), *A_C_* is the amount of curcumin added for loading (mg/mL), and *A_P_* is the amount of copolymer added for loading (mg/mL).

For particle size analysis, 20 μL of CD or CN was diluted with 980 μL deionized water. The particle size and size distribution of the systems were measured according to the method previously described [37] using a Malvern system (Zetasizer ZS, Malvern, UK) working on the principle of dynamic light scattering at a fixed angle of 173 °C.

### 2.3. Cell Preparation

Normal RBCs and PBMCs were collected from the whole blood obtained from healthy donor volunteers at Chiang Mai University, Thailand. The use of human PBMCs and RBCs in this study was conducted in accordance with the Declaration of Helsinki and approved by the Human Research Ethics Unit of the Faculty of Associated Medical Sciences, Chiang Mai University (AMSEC-63EM-009).

For RBCs, the whole blood sample was centrifuged at 2000 rpm for 10 min. The supernatant was removed, and the RBCs were re-suspended with an equal volume of PBS for further study. For normal PBMCs, the whole blood sample was anticoagulated with heparin and diluted with the same volume of PBS. The separation of PBMCs was performed using Ficoll-Paque solution and centrifuged at 5000 rpm for 30 min at 20 °C. The PBMCs were collected, pelleted down, and re-suspended in an equal volume of complete RPMI 1640 medium with 100 unit/mL penicillin, 100 µg/mL streptomycin, and 10% (*v*/*v*) fetal bovine serum. For leukemic cell preparation, cell lines of K562 were cultured in a complete RPMI1640 medium containing 10% fetal bovine serum, 1 mM L-glutamine, 100 unit/mL penicillin, and 100 μg/mL streptomycin and incubated at 37 °C in 5% CO_2_ and 80% humidity.

### 2.4. Effect of CD and CN on Normal Cells

The effect of the samples on normal RBCs was assessed as follows. The prepared RBCs were re-suspended in 8 mL of PBS. Different concentrations of CD or CN (0–100 μM, 100 μL), as well as 1 mg/mL of curcumin in DMSO solution (CM), were incubated at 37 °C with 400 μL of RBCs. After 2 h of incubation, the extent of hemolysis caused by all treatments was observed. The samples were centrifuged at 2000 rpm for 10 min and the supernatant was measured for optical density at 570 and 630 nm using a microplate reader (AccuReader, Metertech-Inc., Taipei, Taiwan). A hypotonic solution of 75 mM KCl was used as a positive control and an isotonic solution of 100 mM PBS was used as a negative control. The percent of hemolysis was calculated using Equation (3):

(3) $$Hemolysis(\%)=\frac{A_{CONTROLE}}{A_{SAMPLE}}\times 100$$

where A_CONTROL_ is the absorbance of the supernatant of RBCs and hypotonic solution sample, and A_SAMPLE_ is the absorbance of the supernatant RBCs and sample.

For the investigation of the effect on normal PBMCs, the cell viability of PBMCs after exposure to the test samples was examined using an MTT assay. Briefly, 100 µL of 1.0 × 10^5^ PBMCs was transferred into a 96-well plate and incubated at 37 °C in 5% CO_2_ and 80% humidity for 24 h. Next, 100 µL of CM, CD, and CN with a curcumin concentration range of 3–100 µM, as well as BD and BN with concentrations ranging from 20 to 600 µg/mL, were added to the cells and incubated under the same condition for 72 h. After that, 100 µL medium was removed and 15 µL of MTT in PBS (5 mg/mL) was added and further incubated for 4 h. After that, the media were removed. The formed formazan crystals were dissolved in 200 µL of DMSO. The absorbance was measured at 570 and 630 nm using a microplate reader.

### 2.5. Effect of CD and CN on Leukemic Cells

K562 cells were cultured in a complete RPMI1640 medium containing 10% fetal bovine serum, 1 mM L-glutamine, 100 unit/mL penicillin, and 100 μg/mL streptomycin and incubated at 37 °C in 5% CO_2_ and 80% humidity. The cytotoxicity of CD and CN in comparison with CM as well as BD and BN on K562 cells was determined using an MTT assay. Briefly, the cells (1.0 × 10^4^ cells/well) were cultured in 96-well plates at 37 °C in 5% CO_2_ and 80% humidity for 72 h. Cell viability was examined by MTT assay, as described in Section 2.4 for PBMCs.

### 2.6. Cellular Uptake of CD and CN to Leukemic Cells

K562 cells (2.5 × 10^5^ cells/mL) were added into a cell culture plate and incubated overnight at 37 °C in 5% CO_2_ and 80% humidity. Then, the cells were added with CM, CD, and CN at a concentration of 20 µM curcumin and further incubated in the same conditions. BD and BN were added to the cells with the same volume as CD and CN, respectively. All treated cells were incubated at 37 °C in 5% CO_2_ and 80% humidity for 4 h. Then, the culture medium was removed. The cells were carefully washed twice with PBS and fixed with 500 µL of cold 2% paraformaldehyde fixative solution for 20 min at room temperature. After the fixative solution was removed, the cells were washed twice with PBS and added to a 96-well glass bottom plate (In Vitro Scientific, Sunnyvale, CA, USA) for investigation under a fluorescence microscope.

### 2.7. Cell-Cycle Analysis

Cell-cycle analysis of K562 was evaluated by flow cytometry using PI which implies the content of the nuclear DNA. K562 cells were treated with the non-cytotoxic concentration at 20% inhibition concentration (IC_20_) of CD, CN, and CM and cultured in culture medium for 72 h. BD and BN were added to the cells with the same volume as CD and CN. PBS and 0.1% DMSO in culture media were used as vehicle controls. The untreated K562 cells were used as cell control to construct a normal distribution for the cell-cycle analysis. After treatment, the cells were washed twice with PBS and prepared as a single-cell suspension. Then, they were fixed with ice-cold 70% ethanol for 30 min. Cell pellets were washed with ice-cold PBS and stained with PI solution in the dark at 4 °C. The red fluorescence was measured using a flow cytometer (Cytomics FC 500, Beckman Coulter Inc., Singapore). The data were analyzed by FlowJo 7.6TM software.

### 2.8. Effect of CD and CN on WT1 Protein Suppression

Western blotting was used in this experiment. K562 cells were treated with CD, CN, and CM at the concentration of IC_20_. Total protein extracts after the treatments were prepared using RIPA buffer (50 mM Tris-HCl, 150 mM NaCl, 1% Triton X-100, 0.5 mM EDTA, 0.1% SDS, and protease inhibitor cocktail) (Sigma-Aldrich, Saint Louis, MO, USA). Protein concentration was measured by the Folin–Lowry method [38]. The protein was separated by 12% SD-SPAGE. The WT1 protein level was assessed using primary rabbit polyclonal anti-WT1 at 1:100 dilutions. Rabbit polyclonal anti-GAPDH at 1:1000 dilutions was used as a loading control. The secondary antibody was a 1:10,000 dilution of goat anti-rabbit IgG conjugated with HRP and used for WT1 and GAPDH protein detection. Proteins were visualized by LuminataTM Forte Western HRP Substrate and exposed to X-ray film. The protein bands were analyzed and quantified by the Quality One Basic 4.6.6 program and a scan densitometer (BIO-RAD, Hercules, CA, USA). The total cell number of K562 after treatments for 72 h was measured by the trypan blue exclusion method.

### 2.9. Statistical Analysis

All experiments were conducted in triplicate and the results were expressed as mean ± SEM. Statistical analysis was conducted by using one-way ANOVA, and the differences were considered significant when the probability value obtained was found to be less than 0.05 (*p* < 0.05).

## 3. Results and Discussion

### 3.1. Preparation of Polymeric Micelles

**Figure 1:**
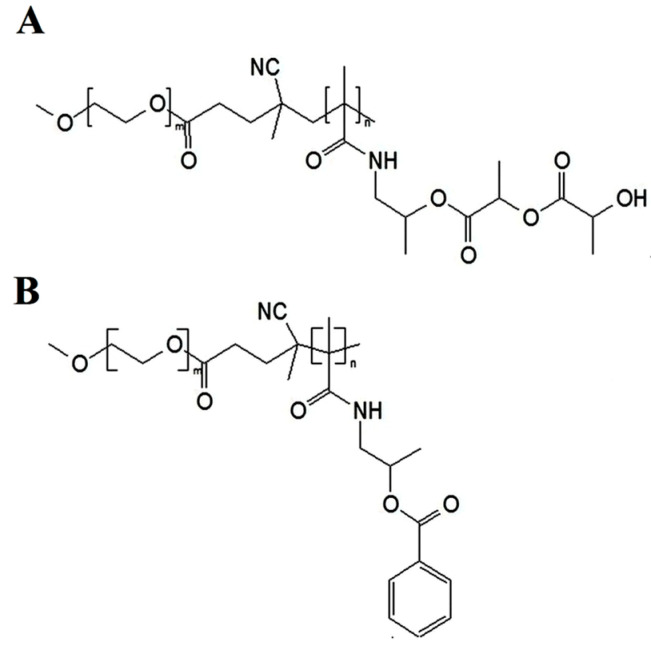
Chemical structure of PEG-HPMA-DL (**A**) and PEG-HPMA-BZ (**B**).

**Figure 2:**
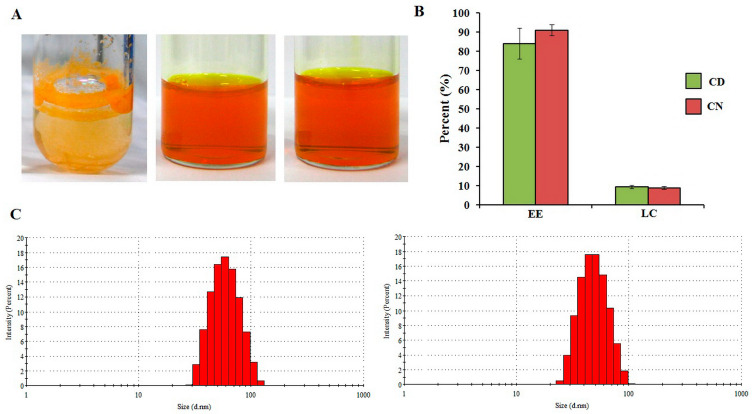
(**A**) Outer appearance of curcumin in water (left), CD (middle), and CN (right), (**B**) percentage of entrapment efficiency and loading capacity of CD and CN, and (**C**) size distribution histogram of CD (left) and CN (right).

According to the structure difference between PEG-HPMA-DL and PEG-HPMA-Bz, as shown in Figure 1, the methods used for the polymeric micellar preparation of these two polymers are different. PEG-HPMA-DL is a thermosensitive polymer due to the lactate content. Its micelles can be formed by heating above the critical micelle temperature, whereas PEG-HPMA-Bz is thermostable, and its micelles can be formed by a nanoprecipitation method. Curcumin is a hydrophobic compound with poor aqueous solubility [10]. It was found that the aqueous solubility of curcumin in CD and CN was greatly enhanced in comparison with the unentrapped curcumin, as shown in Figure 2A. Analysis of curcumin concentration in CD and CN showed different levels of 840 ± 80 µg/mL and 910 ± 30 µg/mL, respectively. Slightly different high drug loading was also found in CD and CN with the entrapment efficiency (EE) of 84 ± 8% and 91 ± 3%, respectively, and the loading capacity (LC) of 8.5 ± 0.8 and 8.0 ± 0.7%, respectively, as presented in Figure 2B. CD and CN exhibited a tiny size of 63 ± 3 and 50 ± 1 nm, respectively, as shown in Figure 2C, with a polydispersity index < 0.2, indicating that the obtained CD and CN possessed narrow size distribution. Both CD and CN colloidal systems observed by visualization showed no precipitation.

### 3.2. Effect of CD and CN on Normal Cells

**Figure 3:**
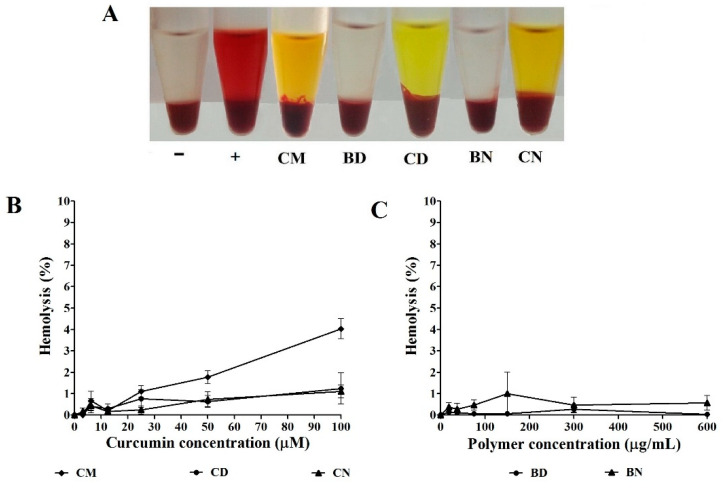
Red blood cell hemolysis after treatment with different formulations in comparison with negative (−) and positive (+) controls (**A**), percentage of hemolysis after treatment with CM, CD, and CB (**B**), and after treatment with BD and BB (**C**).

Compatibility to RBCs or hemocompatibility is important for nanomedicines after administration to the body. The exposure of nanoparticles in the blood circulation should not cause any damage to RBCs or the formation of a thrombus [38]. This experiment was performed to evaluate the hemolysis effect of the test samples. Oxyhemoglobin, which has a dark red color, will leak from the disrupted RBCs after contact with a compound-induced hemolysis [39]. The principle of this assay was to measure the absorbance of oxyhemoglobin at 570 nm. The dark red supernatant of the positive control (hypotonic solution) as shown in Figure 3A indicated the lysis of RBCs, whereas PBS (pH 7.4) as a negative control and all polymeric micelle formulations did not show the color of oxyhemoglobin. The absorbance of all formulations was measured, and the percentages of hemolysis are shown in Figure 3B,C. CM demonstrated obvious toxicity to RBCs. The damage of RBCs caused by CM (4.5 ± 0.1%) was significantly higher than that caused by CD (1.0 ± 0.1%) and CN (1.7 ± 0.1%). The unloaded or blank micelles BD and BN showed low hemolysis of 0.8 ± 1.0 and 0.9 ± 0.6%, respectively. A substance with a hemolytic value below 5% is characterized as hemocompatible according to the critical safe hemolytic ratio for biomaterial, referring to ISO/TR 7406 [40]. The results of this study indicate that the developed CD and CN have strong hemocompatibility, as the hemolysis value is approximately not higher than 3%.

**Figure 4:**
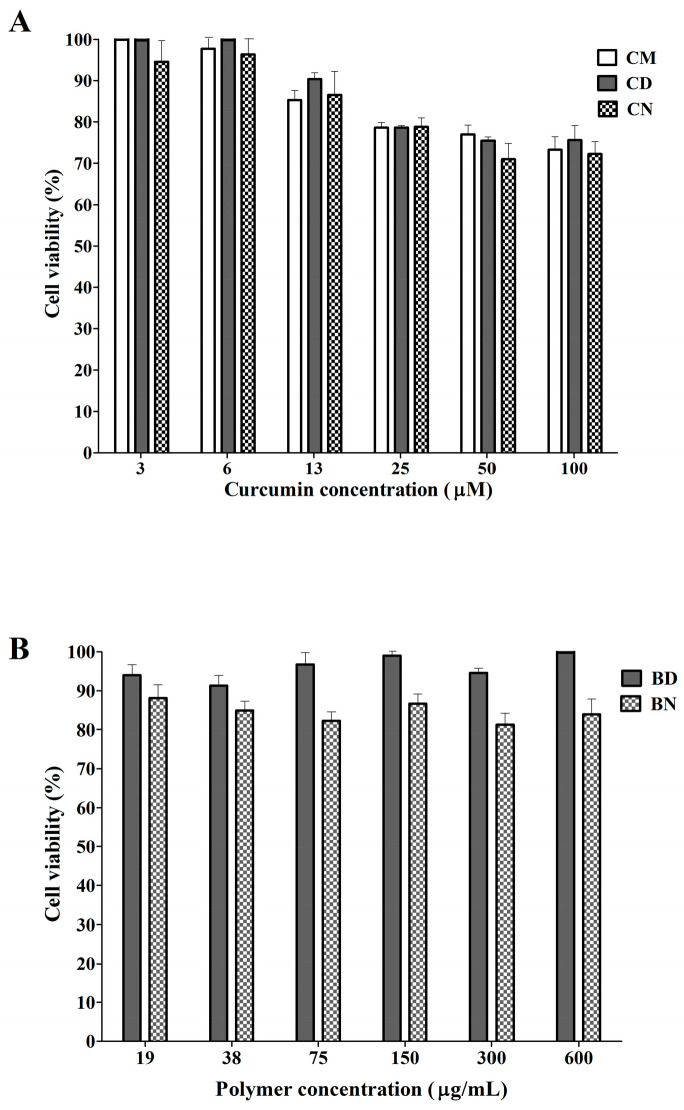
Percentage of cytotoxicity to PBMCs of unloaded curcumin and curcumin-loaded polymeric micelles (**A**) and of blank micelles (**B**).

Cytotoxicity towards normal PBMCs was investigated using a colorimetric method called MTT assay, which is a reaction between MTT and NADPH-dependent cellular oxidoreductase enzymes of the presented viable cells. The high density of purple color indicates a high population of viable cells. This study aims to assess the safety of the test samples. As shown in Figure 4A, normal cell survivability decreased when the concentration of curcumin increased. This dose-dependent manner is found to follow the same pattern among CM, CD, and CN. Generally, with up to 20% cell inhibition, or not less than 80% cell survival, the test sample is considered as safe for normal cells. The results of the present study demonstrate that the test samples containing curcumin up to 25 µM are safe for normal cells. In our previous report [13], we studied the polymer concentration up to 100 µg/mL and found that BD at this concentration was safe for normal PBMCs. In the present work, the studied concentration range of the polymers was increased to higher than 100 µg/mL and the results revealed, interestingly, that the viability of PBMCs after contact with BD was still very high (95 ± 1% of cell viability), even at high concentrations of 600 µg/mL, whereas the cell viability was slightly decreased (81 ± 3% of cell viability) after contact with BN, as seen in Figure 4B. As the cell survival for both BD and BN is higher than 80%, these two polymers are considered as safe for normal PBMCs. The results also suggest that even following modification of the polymeric micelles with aromatic moiety, as for BN, the obtained polymer can be used as safe up to 600 mg/mL. Regarding these results, it can be considered that the developed CD and CN possess good compatibility with normal cells.

### 3.3. Effect of CD and CN on Leukemic Cells

**Figure 5:**
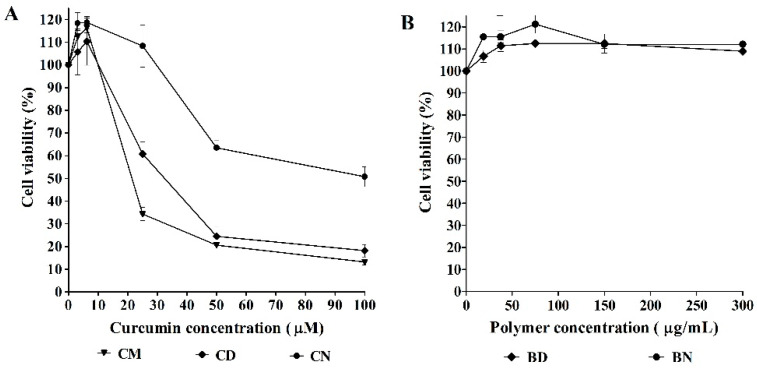
Percentage of cytotoxicity to K562 cells of unloaded curcumin and curcumin-loaded polymeric micelles (**A**) and of blank micelles (**B**).

The results of cytotoxicity of CD and CN in comparison with CM against K562 leukemic cells are shown in Figure 5. It is indicated that BD and BN disclosed good cytocompatibility up to 600 µg/mL, while CD and CN as well as CM showed cytotoxicity to the leukemic cells. The 50% inhibition concentration (IC_50_) of CM (21.5 ± 0.5 μM) is lower than CD (32.2 ± 2.5 μM) and CN (73.8 ± 1.9 μM). However, no significant difference in cytotoxicity is found between CD and CM at the high dose of curcumin (50–100 μM), even though CM displayed a slightly better effect than CD and CN. However, a large amount of DMSO used as a solvent for preparing CM can cause toxicity to normal human cells. Therefore, CM is not suitable for use in humans or animals. The toxicity of curcumin-loaded polymeric micelles against K562 cells is considered to be due to cellular internalization [41,42]. The high IC_50_ of CN is considered to be according to high drug retention in the micelles due to the hydrophobic interaction, the π–π stacking interaction between the aromatic groups of the polymer and curcumin, and the slower intracellular release of curcumin from the polymeric micelles [30].

### 3.4. Cellular Uptake of CD and CN to Leukemic Cells

**Figure 6:**
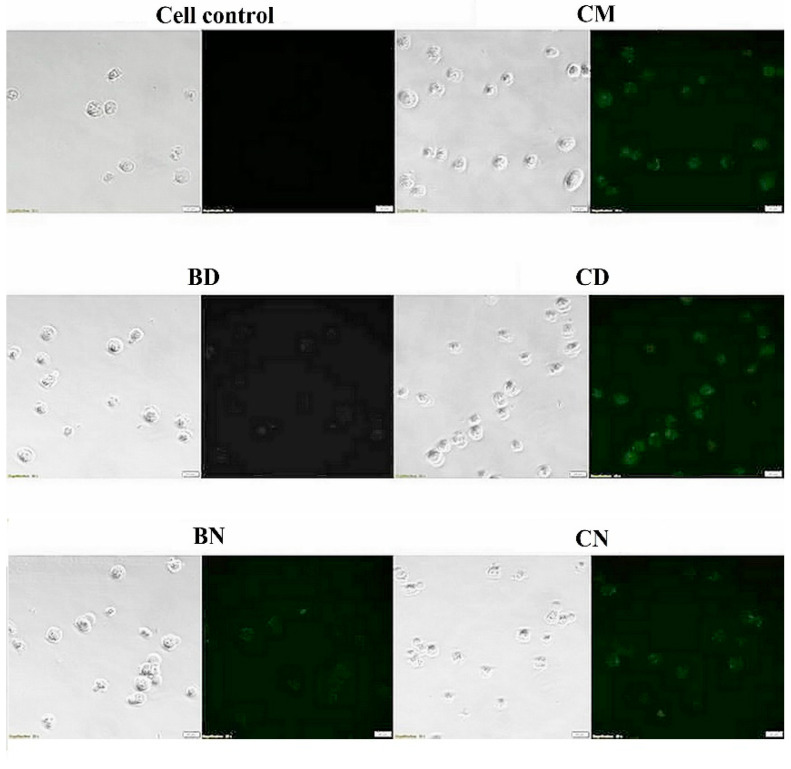
Differential interference contrast (**left**) and fluorescence images (**right**) of internalization by K562 cells of cell control, unloaded curcumin, blank micelles, and curcumin-loaded polymeric micelles.

Curcumin has an advantage in cellular uptake studies because of its natural fluorescence properties. Green fluorescence was observed from curcumin after visualization under the fluorescence microscope. The images of green fluorescence signals inside K562 cells can be seen as shown in Figure 6. No green fluorescence appeared on control cells and BD-treated cells, whereas BN showed slightly green fluorescence, which can be explained by the aromatic content of the polymers that can exhibit fluorescence [43]. CD showed stronger fluorescence intensity than CN after 4 h incubation, indicating that CD was taken up by the cells faster than CN. This result is also possibly due to the slower release of curcumin from CN than from CD. This phenomenon can be explained by the hydrophobic interaction between the aromatic group of curcumin and polymer [34]. The cellular internalization of curcumin-loaded polymeric micelles is likely due to an endocytosis mechanism [44]. Our present results show that modified PEG-HPMA with an aliphatic DL group can be internalized by cells in 1 h and achieve strong fluorescence signals in 24 h. The weaker fluorescence signal of the aromatic BN micelles is considered to be due to the longer cellular uptake and internalization times. In conclusion, our results demonstrate that curcumin-loaded polymeric micelles are effectively taken up by K562 cells, and CD showed this effect significantly better than CN.

### 3.5. Cell-Cycle Analysis

**Figure 7:**
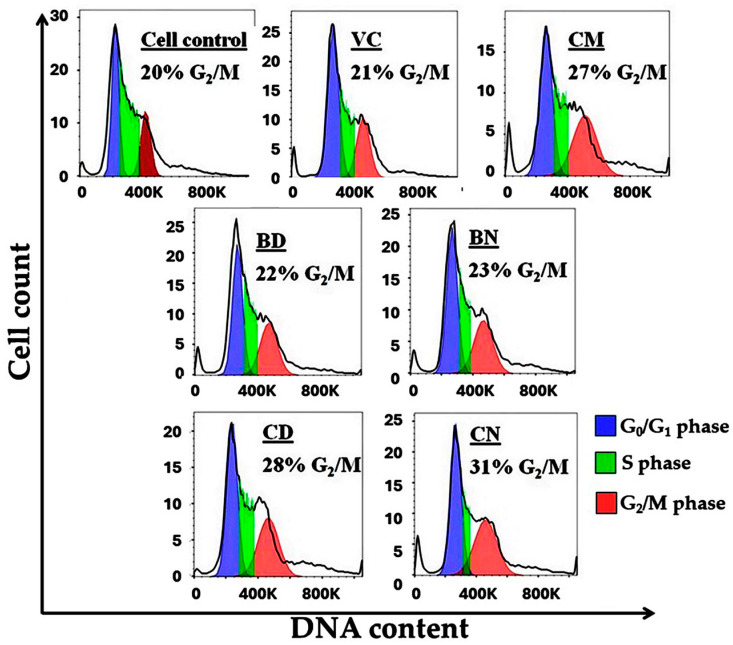
Cell-cycle distribution of K562 cells showing percentage of cells in G_2_/M phase after treating with unloaded curcumin and curcumin-loaded polymeric micelles in comparison with control group. Bars labeled with different letters denote significant difference at *p* < 0.05.

The inhibition of K562 cell growth by curcumin-loaded polymeric micelles was investigated deeply on their effects on cell-cycle progression by using flow cytometry. The non-toxic dose (IC_20_) was used to evaluate cell-cycle arrest in K562 cells and PI was used as a staining dye for DNA content determination. The results of the cell cycle after treating K562 cells with each curcumin formulation are demonstrated in Figure 7. The normal distribution of the K562 cell cycle can be observed in the systems treated with both vehicle controls (PBS and 0.1% DMSO), as shown in Figure 7. BD and BN showed no change in the cell-cycle distribution when compared to the vehicle control. In this study, CM (20 µM) was used as a positive control. It is found that after CM treatment, the cell cycle was arrested at the G_2_/M phase with a value of 27 ± 1.5% as compared to that of the vehicle control. The percentages of G_2_/M populations after treatment with BD and BN are 22 ± 3.3 and 23 ± 3.4%, respectively, while those after treatment with CD and CN were significantly increased with values of 28 ± 3.7 and 31 ± 4.3%, respectively. CM also showed an increase to 27 ± 3.5%. These results indicate that curcumin significantly induces high G_2_/M arrest. According to these results, it can be considered that curcumin loaded in both polymeric micelles possesses a cell-cycle arrest effect as high as free curcumin without any retardation effects of the polymeric carriers. Therefore, it can be concluded that all curcumin-loaded polymeric micelles potentially inhibit the cell-cycle progression of K562.

### 3.6. Effect of CD and CN on WT1 Protein Suppression

**Figure 8:**
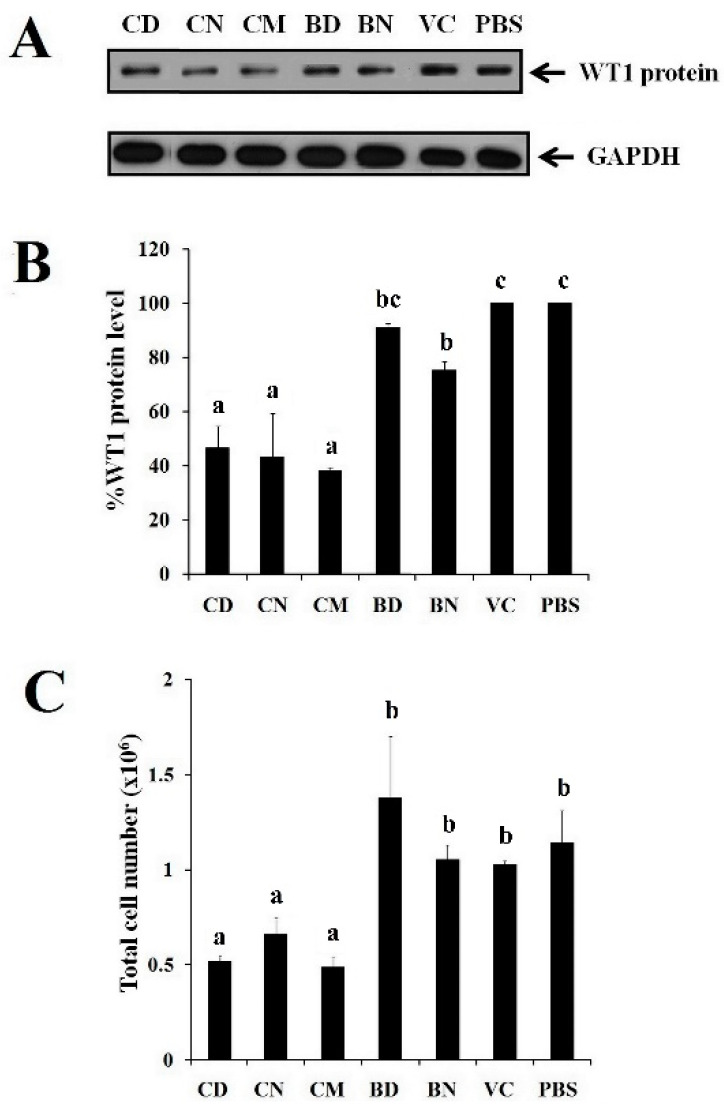
Effect of unloaded curcumin and curcumin-loaded polymeric micelles on the expression of WT1 protein in K562 cells using Western blotting (**A**), percentage of WT1 level quantified by scan densitometer and normalized by GAPDH (**B**), total cell number after treating counted by trypan blue exclusion test (**C**). Bars labeled with different letters denote significant difference at *p* < 0.05.

Overexpression of the WT1 gene plays an important role as a biological marker of leukemia progression [45]. The suppression of the WT1 protein is involved in the G_2_/M phase cell cycle in leukemic cells [46]. Our results indicate that curcumin-loaded polymeric micelles possess cytotoxicity on K562 leukemic cells. They can be taken up by the cells and potentially inhibit the cell-cycle progression. Taking this into account, the comparison of the WT1 protein inhibitory effect of CM, CD, and CN is explored. WT1 protein expression was examined after treating K562 cells with CM, CD, and CN for 72 h. The results as shown in Figure 8 demonstrate the suppression of WT1 protein expressions by CD, CN, and CM in comparison with BD, BN, and the vehicle controls, respectively. The GADPH used as an internal protein control was clearly present in all lanes. It is found that CD and CN obviously suppressed WT1 protein expression levels with suppression values of 46.6 ± 8.1 and 43.2 ± 16.2%, respectively, which were significantly higher than CM (38.04 ± 1.5%) and their control (*p* < 0.05), indicating that the suppressive effect of curcumin-loaded polymeric micelles is higher than free curcumin. BD and BN showed a slightly higher reduction in WT1 protein level expression than the vehicle controls, indicating that the polymers have a slight effect. Furthermore, CD and CN showed a significant decrease in the total cell number of K562 cells by 41.7 ± 0.7 and 42.2 ± 3.6%, respectively, when compared to their control (*p* < 0.05). The results of this WT1 protein suppression are in good agreement with the study of the cytotoxicity of leukemic cells mentioned above which found that curcumin-loaded polymeric micelles strongly reduced the total cell number of K562 cells. This indicates that the proliferation of K562 cells can be potentially inhibited by the developed CD and CN. These results also confirm that WT1 suppression can affect cell proliferation in leukemogenesis. It is considered that curcumin-loaded PEG-HPMA-based polymeric micelles are promising inhibitory systems for the suppression of WT1 expression and K562 cell proliferation.

## 4. Conclusions

The present study demonstrates that highly effective antileukemic water-soluble curcumin can be successfully prepared by loading curcumin into PEG-HPMA-based polymeric micelles. The modification of PEG-HPMA-based polymeric micelles with aliphatic DL and aromatic Bz groups plays a significant role in cytotoxic activity to both normal cells and cellular internalization into the leukemic K562 cells. The micelles modified with DL exhibit better compatibility with normal red blood cells and peripheral blood mononuclear cells than those modified with Bz. The results from the fluorescence microscope indicate that modification with the aromatic Bz group retards cellular uptake and internalization of the micelles. The flow cytometry analysis confirms that curcumin loaded in both micelles possesses a cell-cycle arrest effect on K562 at the G_2_/M phase as high as free curcumin without any retardation effects of the polymeric carriers. The molecular study of the WT1 protein demonstrates that both aliphatic and aromatic-substituted curcumin-loaded polymeric micelles have high suppression activity on this leukemic biomarker protein expression. Comparison between curcumin loaded in the micelle modification with aliphatic DL and aromatic Bz groups allows us to conclude that those modified by the aliphatic substituted group exhibit higher toxicity to the leukemic cells due to their faster uptake and cellular internalization.

## Data Availability

The raw data supporting the conclusions of this article will be made available by the authors on request.
